# MinION Sequencing of colorectal cancer tumour microbiomes—A comparison with amplicon-based and RNA-Sequencing

**DOI:** 10.1371/journal.pone.0233170

**Published:** 2020-05-20

**Authors:** William S. Taylor, John Pearson, Allison Miller, Sebastian Schmeier, Frank A. Frizelle, Rachel V. Purcell

**Affiliations:** 1 Department of Surgery, University of Otago, Christchurch, New Zealand; 2 Biostatistics and Computational Biology Unit, University of Otago, Christchurch, New Zealand; 3 Gene Structure and Function Laboratory, University of Otago, Christchurch, New Zealand; 4 Institute of Natural and Mathematical Sciences, Massey University, Auckland, New Zealand; DuPont Nutrition Biosciences ApS, UNITED STATES

## Abstract

**Background:**

Recent evidence suggests a role for the gut microbiome in the development and progression of many diseases and many studies have been carried out to analyse the microbiome using a variety of methods. In this study, we compare MinION sequencing with meta-transcriptomics and amplicon-based sequencing for microbiome analysis of colorectal tumour tissue samples.

**Methods:**

DNA and RNA were extracted from 11 colorectal tumour samples. 16S rRNA amplicon sequencing and MinION sequencing was carried out using genomic DNA, and RNA-Sequencing for meta-transcriptomic analysis. Non-human MinION and RNA-Sequencing reads, and 16S rRNA amplicon sequencing reads were taxonomically classified using a database built from available RefSeq bacterial and archaeal genomes and a k-mer based algorithm in Kraken2. Concordance between the three platforms at different taxonomic levels was tested on a per-sample basis using Spearman’s rank correlation.

**Results:**

The average number of reads per sample using RNA-Sequencing was greater than 129 times that generated using MinION sequencing. However, the average read length of MinION sequences was more than 13 times that of RNA or 16S rRNA amplicon sequencing. Taxonomic assignment using 16S sequencing was less reliable beyond the genus level, and both RNA-Sequencing and MinION sequencing could detect greater numbers of phyla and genera in the same samples, compared to 16S sequencing. Bacterial species associated with colorectal cancer, *Fusobacterium nucleatum*, *Parvimonas micra*, *Bacteroides fragilis* and *Porphyromonas gingivalis*, were detectable using MinION, RNA-Sequencing and 16S rRNA amplicon sequencing data.

**Conclusions:**

Long-read sequences generated using MinION sequencing can compensate for low numbers of reads for bacterial classification. MinION sequencing can discriminate between bacterial strains and plasmids and shows potential as a cost-effective tool for rapid microbiome sequencing in a clinical setting.

## Introduction

The gut microbiome and its relationship to human health and disease is an area of emerging research. Alterations in microbiome composition have been associated with diarrhoea [1], developmental disorders [2], immune system changes [3], Crohn’s disease [4], psychological disorders [5], irritable bowel disease [6], and cancer [7]. Colorectal cancer (CRC) is associated with high mortality, and the incidence is increasing, globally [8, 9]. Lifestyle and diet are strong risk factors for the disease, and changes in the gut microbiome have been linked to CRC development and pathogenesis in an increasing number of studies [10]. The assessment of microbiomes in a clinical setting is not widely practised. Reasons for this include the lack of access to sequencing technology and clinical training for the interpretation of microbiome sequencing data [11]. Sampling, library preparation, and sequencing are expensive and time-consuming, which makes it less feasible for many applications [12, 13].

Microbiome community composition is most commonly assessed using amplicon sequencing of the bacterial 16S ribosomal RNA (rRNA) gene. This gene is highly conserved in bacteria and contains variable regions that can be used for taxonomic differentiation [14]. Protocols and downstream analysis tools are widely available for 16S analysis [15]. Amplicon-based sequencing can be reliably used to detect genus- and phylum-level differences, but has less power for detecting species- and strain-level differences [16]. Additionally, the technique lacks the ability to analyse plasmids or any genomic region outside of the marker gene, e.g. virulence or antibiotic resistance genes, or variations unique to a species/strain [17, 18].

Meta-transcriptomics using RNA-Sequencing is a powerful tool for interrogating transcribed genes in a sample [19] and can be utilised for microbial classification based on specific RNA transcripts [20]. However, RNA-sequencing is labour-intensive and requires costly reagents and specialised protocols. Currently, bioinformatics analysis of meta-transcriptomics data requires specifically designed software, which is more computationally intensive compared to tools available for human gene-expression analysis [21].

Oxford Nanopore Technologies has developed a small, inexpensive and portable sequencing platform, the MinION, which addresses many of the shortcomings of other available typical next-generation sequencing platforms, such as cost, reagent usage, and analysis bottlenecks, by utilising a non-synthesizing sequencing method [13, 22]. This platform can produce sequencing reads from all genomic material in an environmental or clinical sample, and allows taxonomic classification of elements that may not be detected using marker genes or actively transcribed gene sequencing alone.

To investigate the utility of MinION sequencing for complex microbiome analysis, this study has compared the microbiome component of 11 CRC tissue samples using three different sequencing methods: RNA-sequencing, 16S rRNA amplicon sequencing, and MinION sequencing. The study used Kraken2 for rapid k-mer based assignment of taxa. The aim was to determine the applicability of using multiplexed MinION sequencing as a method for rapid, cost-effective bacterial taxonomic classification of clinical tissue samples.

## Methods

### Patient cohort

Colorectal cancer samples were obtained from treatment-naïve tumours during surgical resection. Study participants gave informed written consent, in compliance with the University of Otago Human Ethics Committee (ethics approval number: H16/037). All the relevant guidelines and regulations were followed during the study. Sample metadata can be found in Table 1.

**Table 1:** Colorectal cancer patient cohort metadata.

Sample	Side	Differentiation	Gender	TNM stage
**1**	Left	Moderate	M	1
**2**	Left	Well	F	2
**3**	Right	Moderate	M	3
**4**	Right	Poor	F	2
**5**	Left	Moderate	M	1
**6**	Left	Poor	F	3
**7**	Right	Well	F	1
**8**	Right	Moderate	F	3
**9**	Right	Moderate	F	3
**10**	Right	Well	F	2
**11**	Right	Poor	F	3

TNM, tumour-node-metastases; M, male; F, female.

### DNA and RNA extraction

Tissue samples were frozen in liquid nitrogen and stored at -80°C post resection, and subsequently transferred to RNAlater ICE^™^ (Qiagen) and stored at -20°C. As described previously [23], nucleic acid extraction was performed on < 20mg of tissue by a single operator in one batch to avoid variation in protocol. Tissue disruption was carried out using a Retch Mixer mill. DNeasy Blood and Tissue Mini Kit (Qiagen) and RNEasy Plus Mini Kit (Qiagen) were used for DNA and RNA extraction, respectively. Quantification of the extracted nucleic acids was carried out using a NanoDrop 2000c spectrophotometer (Thermo Scientific, Asheville, NC, USA) and samples were stored at -80°C.

### 16S rRNA and RNA sequencing

16S rRNA amplicon sequencing (16S-Seq) and RNA-Sequencing (RNA-Seq) data were accessed from publicly available sequence data stored in Sequence Read Archive, study SRP117763 [19]. The data files corresponding to the samples accessed in the current study are given in S1 Table.

16S rRNA libraries were constructed using 20ng of DNA for each sample using primer pairs flanking the V3 and V4 variable regions of the 16S rRNA gene were used (16SF_V3: 5′-TATGGTAATTGGCCTACGGGAGGCAGCAG-3′ and 16SR_V4: 5′-AGTCAGTCAGCCGGACTACHVGGGTWTCTAAT-3′). Using Illumina sequencing adaptors and barcodes, 40 cycles of limited cycle PCR were performed. The Illumina MiSeq platform was used for amplicon sequencing to generate paired-end reads 250bp long.

RNA-sequencing libraries were generated using Illumina TruSeq V2 reagents following ribodepletion using RiboZero Gold. The Illumina HiSeq2000 platform was used to generate paired-end reads 125bp in length.

### MinION library preparation

Eleven DNA samples from CRC tissue, corresponding to those used for 16S rRNA sequencing were used for MinION sequencing (see S1 Table). DNA concentration was recorded using a Qubit^**®**^ 2.0 fluorometer prior to library preparation. A reagent blank (nuclease-free water) was included as a technical control. Size restriction was performed on each of the samples, using 0.45x the volume MagBio High Prep beads. For each sample, 400ng genomic DNA was used, the volume adjusted to 7.5μl with nuclease-free water, and 2.5μl of barcode fragmentation mix was added, as per MinION protocol RBK_9054_v2_revA. The samples were incubated in a thermal cycler at 30°C for 1 minute and 80°C for 1 minute. The barcoded samples were then pooled, and DNA was purified using AMPure XP beads and resuspended in 10 μl of 10 mM Tris-HCl pH 7.5 with 50mM NaCl. Then, 1μl of RAP (Rapid sequencing AdaPtor) were added to the barcoded DNA. The resulting library was loaded onto a single MinION R9.4.1 (106) flow cell and sequenced for 48 hrs.

### Sequence processing

#### 16S rRNA amplicon sequencing

Data were accessed from the publicly available sequence data stored in the Sequence Read Archive, study SRP117763 [24]. In brief, the following steps were taken: short overlapping forward and reverse reads from the same fragments were joined using FLASh v1.2.11 [20], and joined overlapped sequences were trimmed to contain only those reads with a 99.99% accuracy. Minimal length of fragments was kept at 50bp using SolexaQA++ v3.1.15 [21]. Using DADA2, chimeric sequences were removed and amplicon sequence variants were picked to assign bacterial taxonomy from a sequence table using the SILVA132 16S rRNA database (13/12/2017 release) [19]. Commands used can be accessed at the DADA2 GitHub page (https://benjjneb.github.io/dada2, accessed 08/05/2018) and in S1 Data. 16S sequencing data can be accessed at the Zenodo repository [24].

#### RNA-Sequencing

Reads >50bp with an accuracy of 99.9% were retained for analysis. A GRCh38p12 human genome index with RefSeq annotation was generated, and RNA sequences mapped using STAR v2.5.3a [23]. Unmapped reads were converted to FASTQ, sorted, and separated from mapped reads using samtools [24]. Bedtools was used to extract unmapped reads as FASTQ files for use in subsequent taxonomic classification analysis [25]. Singletons and paired datasets were combined post taxonomic classification. Additional genome indices for *B*. *fragilis* Q1F2 and *F*. *nucleatum subsp*. *nucleatum* ATCC 25586 were generated and used for RNA-Seq mapping, using STAR v2.5.3a [23].

#### MinION sequencing

Base-calling and first-pass demultiplexing was performed using Albacore v2.3.3. Sequence quality analysis was performed using NanoPlot 0.16.4 [25]. Porechop v0.2.3 was used to remove barcodes and adaptors, and to verify Albacore demultiplexing. Reads were filtered for quality and length, >Q8 and >120bp, respectively, using FiltLong v0.2.0. The GRCh38p12 human genome index was created using Minimap2 v2.14-r883 [26], to which all MinION reads were mapped. Unmapped read SAM files were extracted and converted to BAM, and then to FASTQ format using samtools v1.9 [27]. Sequencing data can be found in the Zenodo repository [28]. Additional genome indices for *B*. *fragilis* Q1F2 and *F*. *nucleatum subsp*. *nucleatum* ATCC 25586 were generated and used for MinION read mapping, using Minimap2 v2.14-r883 [26].

### Taxonomic classification

#### Taxonomic assignment

Taxonomic classification was carried out on all reads that passed processing filters, and did not map to the human genome. Kraken2 v2.0.6-beta was used for taxonomic classification of all Illumina RNA and ONT sequencing data [29]. The genetic data for constructing the databases were retrieved from the NCBI RefSeq library. The database contained archaea and bacterial taxa complete genomes from the RefSeq NCBI database (S2 Table) and included partial assemblies of selected taxa from a group of species known to be associated with CRC (S3 Table). Results were tabulated and analysed using Pavian [30]. Interactive Genomics Viewer v2.4.18 [31] was used to analyse and visualise alignment of sequencing reads with selected bacterial genomes. For 16S rRNA data, amplicon sequence variants were picked to assign bacterial taxonomy from a sequence table using the SILVA132 16S rRNA database (13/12/2017 release) [19], using DADA2. Commands used can be accessed at the DADA2 GitHub page (https://benjjneb.github.io/dada2, Accessed 08/05/2018) and in S1 Data.

#### Statistics

Spearman correlation analysis was carried out using RStudio (R version 3.6); details can be found in the S1 Data document.

## Results

### Read number and length of sequences for each platform

The average number of raw reads per sample was 13,951,214, 233,193 and 68,534 for RNA-Seq, 16S and MinION sequencing, respectively. After processing the raw reads (quality control and removing reads mapping to the human reference), the per-sample average number of reads was 1,941,172, 141,743 and 15,020 for RNA-Seq, 16S and MinION sequencing, respectively (See Table 2).

**Table 2:** Raw and processed read counts per sample for each platform.

	Raw Reads			Processed/Unmapped		
Sample	16S	RNA	ONT	16S	RNA	ONT
**1**	333335	10210344	53982	176823	157209	12212
**2**	175589	16767600	70967	104310	277886	14184
**3**	210849	11692023	75250	119255	356303	17591
**4**	238258	12414326	50637	133700	4690127	16877
**5**	233536	14196953	68558	129813	259746	15884
**6**	291890	11891786	94040	148406	230809	17449
**7**	173621	18376957	77914	96744	664882	13748
**8**	254700	13680558	97671	145899	775763	24821
**9**	210014	13982612	54786	126141	197291	8280
**10**	334496	15947939	43361	195656	1023419	17952
**11**	69391	14302258	66714	40037	222110	8056

RNA-Seq and 16S rRNA paired-end reads were 250bp and 125bp in length, respectively, giving a 250 and 500bp query maximum for these platforms, while the average MinION read length was 1631bp, giving a genomic query sequence more than three times longer on average; the longest MinION read, at 46,392bp, was 92 times longer than the other platforms.

### Mapping to the human genome

RNA-Seq reads were mapped to the human genome GRCh38p12 using the STAR aligner. An average of 1.9 million unmapped reads remained per sample after mapping, 13.9% of the total. MinION sequencing reads were mapped to a GRCh38p12 index using MiniMap2, and an average of 762 non-human reads remained per sample after mapping, 16.9% of the total. All unmapped reads were putatively bacterial and were used in subsequent classification analysis.

### Comparison of bacterial taxonomic profiles derived using different sequencing platforms

**Fig 1:**
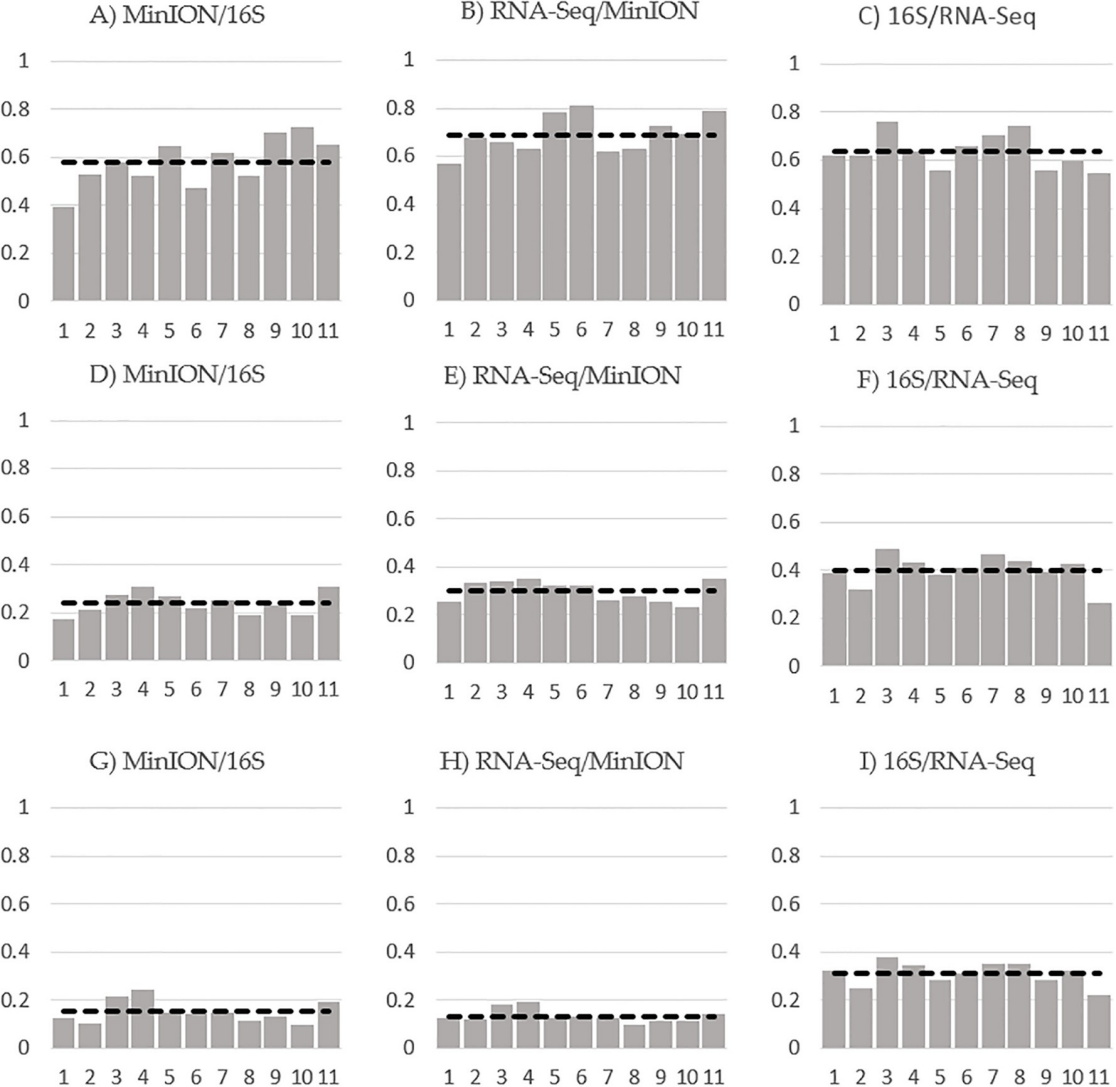
Level of concordance per sample between platforms using Spearman’s rank correlation coefficients of all detected and absent taxa data at A–C) the phylum level, D–F) the genus level and G–I) the species level. The dashed line indicates the average correlation across all samples.

Taxonomic profiles to the species level were generated using Kraken2 for all three sequencing platform data (S4 Table). The concordance between the sequencing platforms (Fig 1) on a per-sample basis was found to be highest at the phylum level between RNA-Seq and MinION. However, at the genus and species levels, the correlation between 16S rRNA and RNA-seq was stronger.

Using Spearman’s rank correlation coefficients to evaluate concordance, we found an average of 0.63, 0.39 and 0.31 concordance between 16S rRNA amplicon sequencing and RNA-Seq at the phylum, genus and species levels respectively. Concordance between RNA-Seq and MinION was 0.68, 0.29 and 0.13 at the phylum, genus and species levels, respectively, and concordance between 16S rRNA sequencing and MinION sequencing was 0.57, 0.23 and 0.19 at the phylum, genus and species levels respectively, per sample.

The taxa identified at each taxonomic level from all 11 samples were substantially different between sequencing platforms, ranging from 80.5% similarity in identified phyla to only 18.9% similarity at the species level between 16S rRNA amplicon sequencing and RNA-Seq (Table 3).

**Table 3:** Similarity in taxa identified between platforms.

	16S v RNA-Seq	16S v MinION	MinION v RNA-Seq	16S v RNA-Seq v MinION
**Phyla**	80.50%	67.60%	66.70%	59.50%
**Genus**	36.70%	35.80%	51.50%	23.30%
**Species**	18.90%	19.50%	35%	9%

### Microbiome sample composition

**Fig 2:**
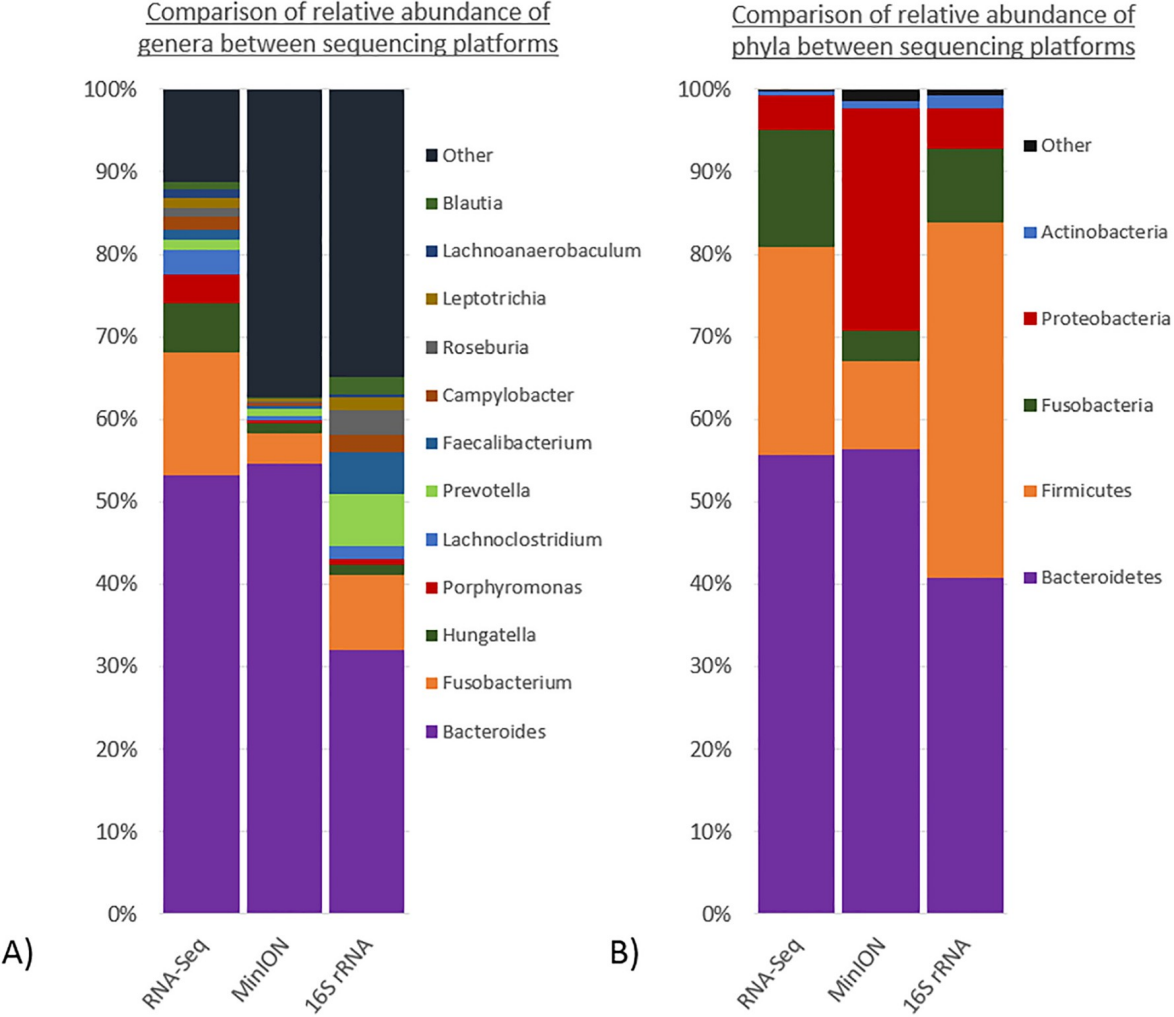
Comparison of relative abundance of between sequencing platforms at A) the phylum level and B) the genus level.

To estimate the relative abundance of taxa within samples using Kraken2, we used the number of assigned reads as an indicator of an individual bacteria. Fig 2A shows the proportional composition detected using each of the platforms, as estimates of abundance of different taxa. The relative abundance of phyla detected using each platform varied, with 16S rRNA sequencing detecting higher levels of Firmicutes than Bacteroidetes, compared to the other platforms, while a larger proportion of MinION sequencing reads were assigned to Proteobacteria. As expected, Firmicutes, Bacteroidetes, Fusobacterium and Proteobacteria were the most abundant phyla detected, although their relative abundance differed depending on the sequencing platform used.

The relative abundance at the genus level also differed substantially between sequencing platforms, as shown in Fig 2B. A lower proportion of *Bacteroidetes* and a high proportion of *Prevotella* was detected using 16S rRNA sequencing. RNA-Seq detected a significantly larger proportion of *Fusobacterium Hungatella*, and *Porphrymonas* compared to the other platforms.

**Fig 3:**
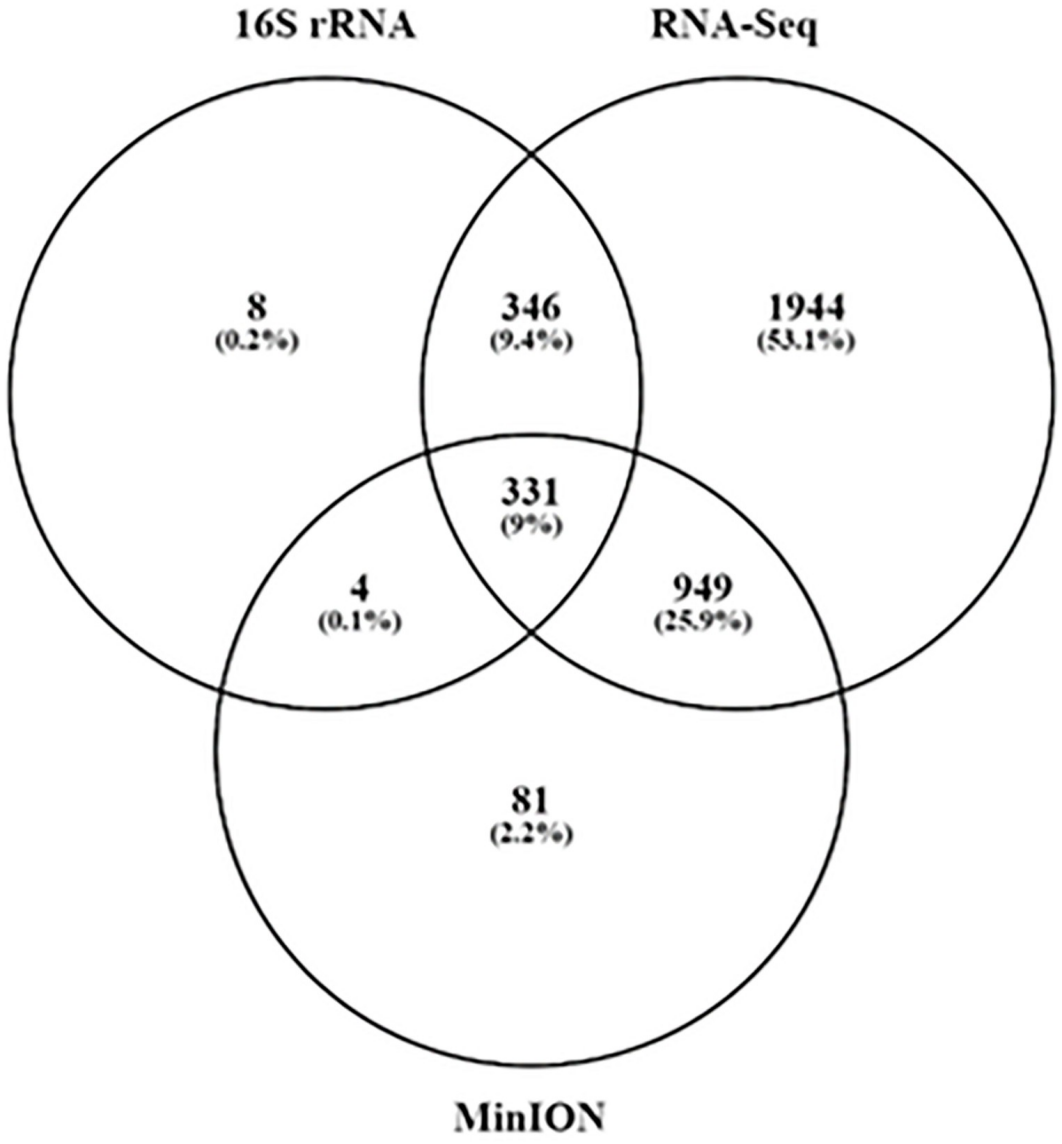
Comparison of bacterial species detection between each sequencing platform.

At the species level, RNA-Seq was able to detect a greater number of species than the other two platforms, while MinION sequencing could detect more than 80 species undetected by RNA-Seq or 16S sequencing. Only 689 species were detected using 16S rRNA sequencing, almost all of which were detected using at least one of the other platforms (Fig 3). The number of reads needed to have sufficient evidence for a species is dependent on the read length of the query [32], and, therefore, the number of raw reads required to classify species is much higher for short-read sequencers. MinION was able to detect one species on average every 893 reads, while 16S rRNA amplicon sequencing required 3724 per species, and RNA-Seq performing most poorly, assigning a single species for every 42,987 reads (Table 4). In this sense, MinION sequencing had an 11.5- and 3.7-fold increase in efficiency over RNA-Seq and 16S rRNA amplicon sequencing, respectively.

**Table 4:** Number of different taxa detected using each sequencing platform.

	RNA-Seq	16S rRNA	MinION
**Phyla detected**	41	33	29
**Genera detected**	1156	424	605
**Species detected**	3570	689	1365
**Unique phyla**	5	0	1
**Unique genera**	405	0	6
**Unique species**	1944	9	81
**Reads per species**	42,986	3723	893

### Detection of colorectal cancer-associated bacterial species

Several bacterial species implicated in CRC were detected across all platforms, such as *B*. *fragilis* [10], *F*. *nucleatum* [33] and *Prevotella intermedia* [34]. Of the 81 species detected only by MinION sequencing (Table 4), several were closely related *Bacillus*, *Burkholderia*, *Streptomyces* and *Pseudomonas* species. A total of nine species were detected only by 16S rRNA amplicon sequencing, with *Sphingobium sp*. YG1 being the most abundant (S4 and S5 Tables). RNA-Sequencing was able to detect 1944 species not detected by any other platform (Table 4), including 49 *Candidatus sp*, and many species yet to be cultured. In addition, RNA-seq and MinION could detect several commensal species, such as *Lachnoclostridium sp* known to be involved in gut health [35], which were not detected using 16S rRNA amplicon sequencing. Across all platforms, *B*. *fragilis* was the most abundant species, followed by *Hungatella hatheway*, *F*. *nucleatum*, *B*. *vulgatus* and *Faecalibacterium prausnitzii* (S4 Table).

### Alignment of reads to colorectal cancer-associated bacteria

**Fig 4:**
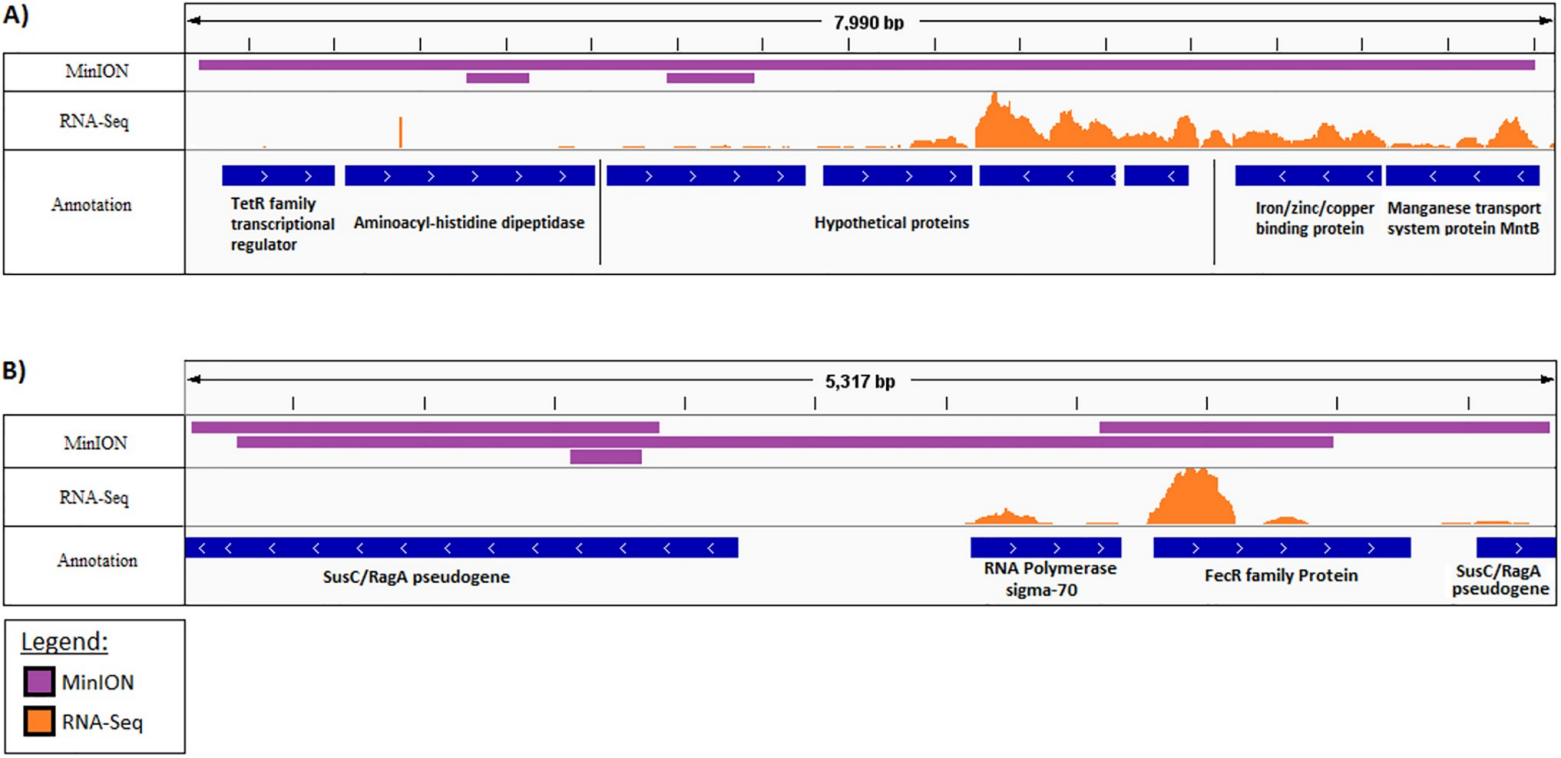
Alignment of MinION and RNA-Sequencing data to bacterial genomes. A) Mapping to *F*. *nucleatum subsp*. *nucleatum* ATCC 25586 genome. B) Mapping to *B*. *fragilis* Q1F2 plasmid. MinION reads are represented in purple and RNA-Seq reads in orange.

After bacterial identification, we wished to evaluate which regions of the genome MinION and RNA-Seq reads would align to. MinION and RNA-sequencing reads were aligned to the genome of *F*. *nucleatum subsp*. *nucleatum* ATCC 25586, which has been associated with CRC [33, 36]. A single 9 kb MinION read covered a region containing four coding genes, including those coding for a putative *TetR* transcriptional regulator, involved in antibiotic resistance, and for an amino-histidine dipeptidase. Separated by four hypothetical protein-coding genes, the mapped region also contained a metal-binding protein gene and manganese transport gene. RNA-Seq reads predominantly mapped to these latter two genes, with few reads mapping to the region downstream of the hypothetical protein genes (Fig 4A). Additionally, sequencing reads were aligned to a conjugative plasmid of *B*. *fragilis* strain Q1F2 (Fig 4B). Four MinION reads, of lengths 4200, 1913, 1760, and 308bp, mapped to the plasmid to cover a 5317bp region. RNA-Sequencing had 75 reads align at the highest peak, aligning to a total region of 1557bp. The majority of RNA-Sequencing reads mapped to the promoter region of a *FecR* iron transport gene and a corresponding sigma-70 polymerase gene.

## Discussion

Microbiome studies are increasingly being carried out on clinical samples and are expanding our knowledge of the involvement of the gut microbiome in human health and disease. Although microbiome analysis is not currently used in the clinical setting, it is likely, with the advent of microbiota-based therapeutics and prognostication, that this type of analysis will be co-opted for clinical use in the near future. The majority of published studies to date have used 16S rRNA amplicon sequencing to describe the structure of the gut microbiome. This has proven a useful tool in determining large-scale changes in the microbiome but using a region of a single gene has less utility in detecting subtle changes in microbial composition at the species level in comparison to metagenomic sequencing, as 16S rRNA amplicons are often too similar at the sequence level to differentiate between species [37]. Metagenomic analysis using shotgun sequencing allows for a more in-depth analysis of the microbiome to the species or even strain level, in addition to capturing mobile genetic elements and plasmids that would not be detectable using amplicon-based approaches. A recent shift towards identifying changes in microbiome function, in addition to composition, has encouraged meta-transcriptomics approaches to microbiome analysis. However, both metagenomics and meta-transcriptomics analyses generally involve short sequencing reads and necessitate high read-depth for adequate genome coverage and accurate alignment.

The Oxford Nanopore MinION is a portable sequencing device that has recently emerged as a rapid and cost-effective sequencing platform that produces long reads, and has multiple applications in clinical microbiology, such as pathogen detection [38], bacterial genome assembly [39] and plasmid and resistance gene detection [40, 41]. Specific to microbiome analysis, the utility of the MinION platform has been demonstrated in rRNA amplicon-based sequencing [42–44] and in metagenomics for outbreak analysis of bacteria [45] and viruses [46].

In this study, we compared MinION sequencing to two currently used microbiome analysis platforms, 16S rRNA amplicon sequencing and meta-transcriptomics using RNA-Sequencing. The analysis was carried out using heavily host-sequence contaminated RNA/DNA extracted from colorectal tumour tissue samples and no attempt was made to reduce the amount of host genomic material in any of the extraction methods; only 16S rRNA sequencing samples selected specifically for microbial DNA.

For MinION sequencing, we barcoded the 11 samples and ran them as a multiplexed library on a single flow-cell at a cost of approximately US$1000, a fraction of the cost of 16S rRNA and RNA-Sequencing. Labour and capital costs are very important considerations for pilot studies, and in particular for studies where samples are limited. As there was no amplification step involved, the numbers of reads were several orders of magnitude lower than 16S sequencing or RNA-Seq. Following a demultiplexing step, the available putative bacterial reads per sample were as low as 8056 for some samples. Despite this, the most abundant organisms could still be detected in these samples. One contributing factor to the low numbers of post-mapping reads was that almost half of the sequenced reads were not barcoded, which, in addition to reducing the power of the analysis, may introduce a sampling bias. Additionally, the lower number of reads generated by MinION sequencing reduces its capacity to detect rare taxa present at low abundance in samples. An enhanced library preparation protocol may increase the overall fraction of barcoded reads. Multiplexing of samples also meant that the DNA input per sample was relatively low. Sequencing a single sample or fewer multiplexed samples per flow-cell would increase the overall number of acquired reads per sample. Since the inception of this study, due to improvements in library preparation chemistry, software and improved flow-cell manufacturing, the throughput and basecalling accuracy of Oxford Nanopore MinION sequencing has increased, with higher numbers of reads of consistent quality and length being produced, widening its applications and reliability of results [47–50].

We used Kraken2 [29] for rapid taxonomic identification of MinION and RNA-Seq data, with a customised database that included many additional CRC-associated taxa. Kraken2 uses a k-mer based algorithm that uses fragmented whole genomes as the basis of taxa classification [51], without requiring large amounts of computational resources, and with increased speed compared to direct alignment of genomic sequences, such as BLAST [52].

Concordance between 16S rRNA sequencing and the other two platforms ranged from 59.5–80.5% at the phylum level but was as low as 9% at the species level. This reflects the reduced ability of 16S rRNA sequencing to differentiate between species. Concordance between RNA-Seq and MinION sequencing was 66.7% 51.5% and 35% at the phylum, genera and species levels, respectively. The relatively low concordance between RNA-Seq and MinION sequencing can be attributed to the considerably higher number of RNA-Seq reads.

However, despite the low numbers of reads acquired using MinION sequencing, more than a 1300 species could be assigned taxonomy, the majority of which were also detected in the corresponding RNA-Seq data but not using 16s rRNA. Specific analysis of long, high-quality MinION reads demonstrated that MinION’s longer reads can compensate for a lower number of reads, as it is possible to observe more inter-microbial genomic complexity giving a higher resolution taxonomic assignment. This has been demonstrated by Wommack et al., who found that long reads could detect 72% more hits than short read lengths of up to 400bp at twice the read depth [32]. Although Oxford Nanopore long-read sequencing is acknowledged to have an inherently high error rate, longer read length has been shown to compensate for this [53].

Comparison of the three sequencing platforms at different taxonomic levels showed that at the phylum level, all three platforms could detect a core set of common phyla, which included the most common gut-associated phyla from the Human Microbiome Project [54]. RNA-Seq was able to detect five phyla that were not detected in the MinION data, reflecting the high numbers of reads achieved with that sequencing approach, but may also be attributed to the non-synthesis-based method of sequencing MinION employs that may influence which sequences are analysed by a pore, introducing a possible selection bias. Additionally, the higher performance of RNA-Seq compared to MinION sequencing could be due to RNA-Seq transcripts being more likely to be found in microbial genome databases, leading to a higher likelihood of detection and improved estimates of relative abundance [55].

Although the concordance between MinION and RNA-Seq was high at the phylum level, the overall taxonomic assignments at the species level were considerably lower, likely due to the reduced numbers of reads observed using MinION sequencing. Mapping of RNA-Seq and MinION reads to the most recent complete genome of *B*. *fragilis*, showed alignment to plasmids, including genes that code for a putative iron transporter protein, a nutrient thought to be involved in *B*. *fragilis* virulence [56]. Additionally, MinION reads mapped to a region of the *F*. *nucleatum* genome which contains a putative antibiotic resistance gene. These results suggest that MinION sequencing can be informative for the analysis of regions of interest, such as antibiotic resistance genes and mobile elements [57]. Using a more refined protocol, it would be feasible to screen an entire plasmid using single long reads, or low read depth to detect pathogenicity and antibiotic resistance genes. The RNA-Seq platform likely did not detect the genes associated with antibiotic resistance as they were not being actively transcribed at the time. This highlights the importance of choosing the appropriate sequencing platform or combination of platforms to suit the experimental conditions.

## Conclusions

Here, we have shown that direct microbiome sequencing from CRC tumour samples is feasible using the MinION platform, and gives high taxonomic concordance compared to RNA-Seq, and may be superior to 16S rRNA sequencing for taxonomic classification to the species level. We have shown that long-read sequences can compensate for low read depth for classification purposes. Our investigation has also shown the ability of MinION sequencing to discriminate between bacterial strains and detect bacterial plasmids, and shows potential as a tool for cost-effective microbiome sequencing in a clinical setting.

## Supporting information

S1 Data (DOCX)

S1 Table (XLSX)

S2 Table (XLSX)

S3 Table (XLSX)

S4 Table (XLSX)

S5 Table (XLSX)
