# Supplementary material for: MinION Sequencing of colorectal cancer tumour microbiomes—A comparison with amplicon-based and RNA-Sequencing
Source: PLoS One. 2020 May 20;15(5):e0233170. doi: 10.1371/journal.pone.0233170 (PMC7239435; doi:10.1371/journal.pone.0233170)
Supplement: S1 Data — (DOCX) [file pone.0233170.s001.docx]

**Supplementary Methods**

Commands and parameters used for respective software.
**STAR**

#index generation

STAR --runThreadN 12 --runMode genomeGenerate --genomeDir genomes/ --genomeFastaFiles genome.fna

#For bacterial genomes the additional parameter was used:

--genomeSAindexNbases 11

#mapping

STAR --runThreadN 12 --genomeDir genomes/ --readFilesIn input.r1.fastq input.r2.fastq --outSAMunmapped Within --outFileNamePrefix .mapped

**Samtools**

#Converting .sam to .bam files

samtools view -bS in.sam > out.bam

#extracting unaligned reads from bam files

samtools view -b -f4 bammed.bam < unmapped.bam

#sorting unmapped bam files

samtools sort -n unmapped.bam -o unmapped.sorted.bam

**Bedtools**

#MinION reads

bedtools bamtofastq -i input.sorted.bam -fq output.fastq

#paired end rna-seq reads

bedtools bamtofastq -i input.sorted.bam -fq output_r1.fastq -fq2 output_r2.fastq

**Albacore**

Albacore -t 12 -f FLO-MIN106 -k SQK-RBK004 --recursive --barcoding -n 0

**Nanoplot**

Nanoplot --summary sequencing_summary.txt -loglength -o summary-plots-transformed

**Porechop**

porechop -t 12 --middle_threshold 99.0 --barcode_diff 1.0 --barcode_threshold 75.0 --format fastq -i /input_dir/ -b /output_dir/

**FiltLong**

filtlong --min_length 120 --min_mean_q 8 input.fastq > output.filtered.fastq

**MiniMap2**

#database creation

minimap2 -k 28 -w 15 -d genome-index.mmi target_genome.fna

#mapping

minimap2 -ax map-ont genome-index.mmi input.fastq > output.bam

**Kraken**

#Downloading database items into library

kraken2-build --download-library bacteria --db database

kraken2-build --download-library archaea --db database

find additional_genome_dir/ -name '*.fa' -print0 | xargs -0 -I{} -n1 kraken2-build --add-to-library {} --db database

kraken2-build –-build –-db database

#taxonomic assignment

kraken2 --db database --use-names --report-zero-counts --thread 12 input.fastq --report input.fastq.report > input.fastq.kraken_output

**Statistics**

Spearman’s correlation matrices used for plots in Figure 1 were generated using the cor command using R v 3.6.0 as follows:

X_vs_Y_comparison <- cor(X, Y, method='spearman')

**DADA2 Parameters**

DADA2 commands used can be access in full at: <https://benjjneb.github.io/dada2>

Filtering:
truncLen=c(240,160),

maxN=0, maxEE=c(2,2), truncQ=2, rm.phix=TRUE,

Error rates were calculated using the learnErrors command for forward and reverse reads, rates were plotted for confirmation using the plotErrors command.

Sample inference was performed using the dada command and paired reads were merged using the mergePairs command on the resulting dada2 object.

Sequence table was constructed using makeSequenceTable from the merged reads. Chimeras were removed from the data using the removeBimeraDenovo command using the “consensus” method.

Taxonomy was assigned using the assignTaxonomy command and the Silva v132 training set. Species level assignment was performed using the addSpecies command and the silva_species_assignment_v132 data from the silva 132 database. (The reference files can be found here <https://benjjneb.github.io/dada2/training.html>).

The resulting taxonomy was converted to a dataframe for export and comparative analysis.
